# Defining Soft Tissue: Bitmap Printing of Soft Tissue for Surgical Planning

**DOI:** 10.1089/3dp.2021.0141

**Published:** 2022-12-13

**Authors:** Nicholas Jacobson, Erik Carerra, Lawrence Smith, Lorna Browne, Nicholas Stence, Alison Sheridan, Robert MacCurdy

**Affiliations:** ^1^ School of Engineering, Design and Computation—Inworks Innovation Initiative, University of Colorado: Anschutz Medical Campus, Aurora, Colorado, USA.; ^2^ School of Engineering, University of Colorado: Boulder, Boulder, Colorado, USA.; ^3^ Pediatric Radiology, School of Medicine, University of Colorado: Anschutz Medical Campus, Aurora, Colorado, USA.; ^4^ School of Medicine, University of Colorado: Anschutz Medical Campus, Aurora, Colorado, USA.; ^5^ Department of Pediatric Neuroradiology, Children's Hospital Colorado, Aurora, Colorado, USA.; ^6^ Mechanical Engineering, University of Colorado: Boulder, Boulder, Colorado, USA.

**Keywords:** biomedical, multimaterial, bitmap, medical imaging, MRI

## Abstract

Nearly all applications of 3D printing for surgical planning have been limited to bony structures and simple morphological descriptions of complex organs due to the fundamental limitations in accuracy, quality, and efficiency of the current modeling paradigms and technologies. Current approaches have largely ignored the constitution of soft tissue critical to most surgical specialties where multiple high-resolution variations transition gradually across the interior of the volume. Differences in the scales of organization related to unique organs require special attention to capture fine features critical to surgical procedures. We present a six-material bitmap printing technique for creating 3D models directly from medical images, which are superior in spatial and contrast resolution to current 3D modeling methods, and contain previously unachievable spatial fidelity for soft tissue differentiation. A retrospective exempt IRB was obtained for all data through the Colorado Multiple Institution Review Board #21-3128.


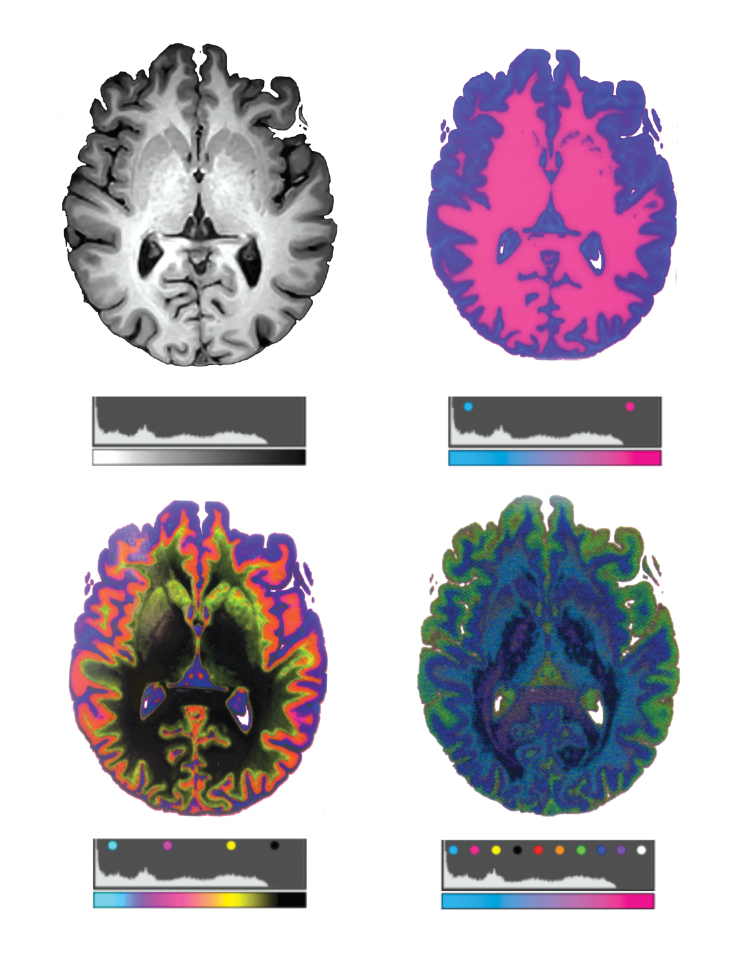


## Introduction

The needs of the biomedical industry to describe soft tissue, which exhibits gradually varying material distributions across multiple domains, call for advanced methods of physical representation. Considering that medical images of soft tissue typically capture hundreds to thousands of unique volumetric density values representing a given anatomical structure, the predominant surface mesh-based 3D printing formats become incapable of fully representing these data. Furthermore, the explicit nature of this surface-based modeling paradigm has proven inadequate for expressing the granular or hierarchical structure of complex human organs, which contain sui generis scales of organization. The dominant representational framework that digital medical modeling tools employ today for 3D printing utilizes a boundary representation file format, which requires one discrete material to occupy the interior of the boundary as solid, homogeneous, isotropic materials.^1^

Recent advancements in 3D printing file formatting, which include image-based surface mapping and vertex-based color gradients, have shown promise for printing gradients, but they still lack the ability to accurately replicate volumetric variations in material distribution.^2^ As a result, 3D printing for presurgical planning has been mostly limited to bony structures and limited morphological descriptions of complex organs.^3^ Bitmap printing has recently allowed the direct fabrication of 3D printed models from medical images.^4^ This article quantifies a few of the benefits of bitmap printing over the predominant mesh-based boundary surface representation file format for studying local differences in soft tissue replication.

In recent years, an increased clinical interest has emerged in transcending morphological structures (form) to include mechanically functional materials, which replicate soft tissue behaviors for passive tangible haptic feedback (function) in preparation for surgical cases.^4–6^ Three-dimensional printing has emerged as a new diagnostic tool for presurgical planning, however, it has not yet taken full advantage of the data captured in radiological imaging, ignoring graduated morphological and radiodensity data.^7^ One of the limitations facing the complex high-resolution digital fabrication required to accurately 3D print tissue comes from the fundamental inability of the predominant digital fabrication tools to represent spatial variations in material properties.^8^ Another limitation is a lack of understanding of the levels of refinement required to represent unique soft tissue structures through bitmap printing. The ability to accurately translate medical images to 3D printed models at the resolution of the source data would improve the ability of clinicians to better plan for complex surgeries related to soft tissue and better communicate with their patients and extended medical team.

Bitmap printing has emerged as a new method of additive manufacturing and produces 3D prints directly from medical images at a level of spatial fidelity and spatial contrast resolution equivalent to the source images.^9^ Previous bitmap printing attempts have been limited to using two materials to replicate variations in high resolution.^9^ However, two materials limit the ability to clearly define differences across an organ defined by numerous different anatomical descriptions. We have created a six-material bitmap printing process, which simplifies the current bitmap printing process and more clearly defines differences in tissue variation.

To show the strength of our method, we focus on the translation of radiodensity information from magnetic resonance imaging (MRI) to a 3D print applied to three different anatomies: an adult kidney, an adolescent brain, and a pediatric heart. We study the emergence of anatomical features specific to each organ over the sequential incorporation of two, four, and six unique printable materials. Next, we compare the predominant mesh-based boundary surface model 3D printing method to our six-material bitmap printing method. These tests show the boundary representation process to be wasteful in file size, cumbersome due to the increased number of discrete files, and low resolution, which leads to inaccuracies and volume loss. In contrast, models fabricated using our high-resolution bitmap printing method are derived directly from high-resolution medical images with comparable spatial contrast resolution. Finally, as a preliminary pilot comparison study, we blindly presented a board-certified Radiologist and Surgeon, in each related specialty, with both sets of models and a qualitative survey. These results showed an overwhelming preference for our bitmap method.

In this article, we demonstrate that a six-material bitmap-based 3D printing process is more suited for accurately replicating soft tissue from radiological images than the predominate boundary representation method and more recent two-material bitmap methods. The benefits of this workflow include (1) a simplified method for multimaterial bitmap printing, (2) precise graduated control over the material distribution at multiple scales within a 3D printed volume, and (3) a more accurate representation of anatomical features in soft tissue.

## Methods

Three soft tissue anatomies were studied: adult kidney, adolescent brain, and pediatric heart. All imaging was deidentified and not used clinically, therefore the requirement for individual informed consent was waived. Digital Imaging and Communications in Medicine (DICOM) data were acquired from new multidetector MRI. Each model consisted of data solely from one imaging session and specific protocols were chosen for printing with the guidance of Board-Certified Radiologists in their respective disciplines of Body Imaging, Pediatric Cardiology, and Pediatric Neurology.

### Method 1—Bitmap-based workflow

**FIG. 1.**
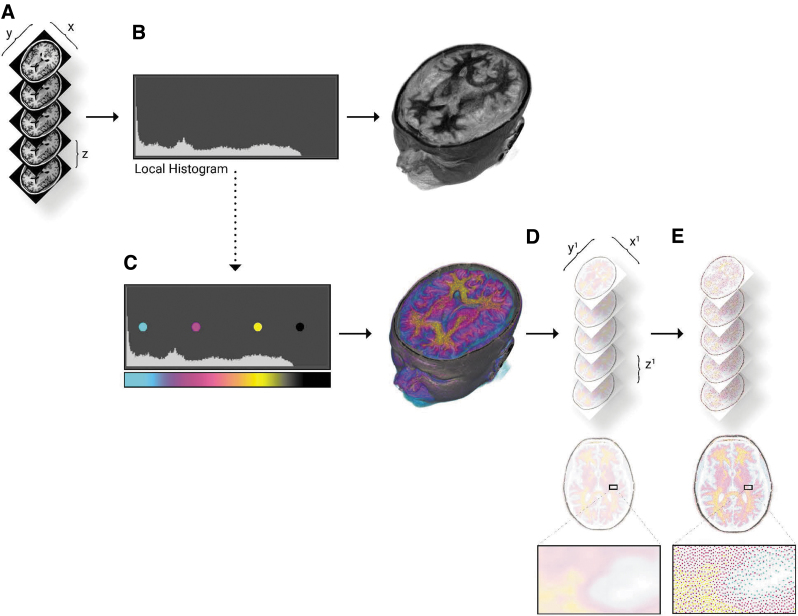
Bitmap processing workflow for modeling radiodensity. **(A)**The input DICOM data are loaded at the resolution of the scanner, the highest *x*, *y* resolution, and thinnest *z* slice thickness results in more detailed final models. **(B)** A histogram of the loaded DICOM intensity values is analyzed to parse the ranges of intensity values. If left unmodified, a *black* and *white* voxel representation will be created. **(C)** The material channel of the voxel-based volume rendering is modified, through lookup tables, which map color to the specified intensity ranges. **(D)** The combined volume rendering is sliced as full-color PNG files to the required constraints and resolution of the printer. **(E)** Every PNG slice is dithered into each material description that is needed to fabricate the data. The resulting stack of colored and dithered PNG files send directly to the printer for fabrication. DICOM, Digital Imaging and Communications in Medicine.

**FIG. 2.**
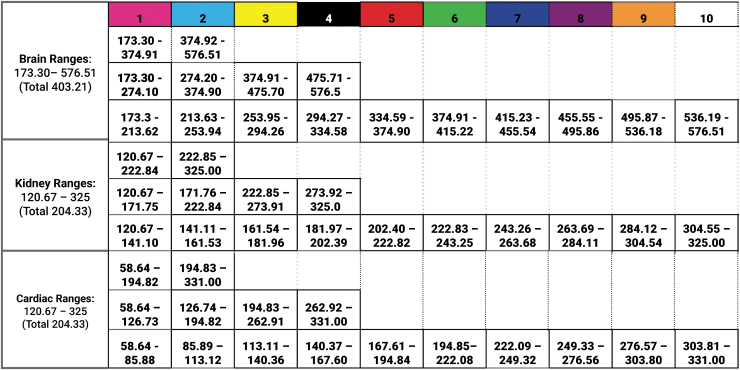
Radiodensity divisions. This chart depicts the list of Hounsfield values studied for each masked and isolated anatomical area. The subsequent 2, 4, and 10 segmentations equally divided the full range of values for the entire masked and isolated anatomical area.

An overview of our first method is shown in Figure 1. DICOM files from each anatomy are first processed with the open-source 3DSlicer software package.^10^ It is critical to select imaging sequences that contain the highest resolution images with the thinnest slice acquisition since this method is capable of printing at 15 and 27 μm slice thickness, depending on the print mode. Window/level adjustments and histogram processing is completed similar to Hosny *et al.*^9^ to emphasize a wider range of the displayed intensity values. After the desired range of values is displayed, the anatomy of interest is cropped and all data, except the anatomy of focus is masked. The remaining field of values constituting the domain of the isolated organ is calculated to find the total bounds. The resulting intensity range is evenly partitioned into sets of discrete values (Fig. 2). A custom lookup table is created to specify the mapping of DICOM intensity values to colors in a voxel-based volume rendering. Smooth gradients are then created between regions of distinct color, relating to the specified threshold ranges, by customizing the computational color blending algorithms of the histograms and lookup table arrays.

Once the color has been mapped to the intensity values of the selected imaging sequence, a voxel-based volume rendering is created by using pixel shaders that implement a ray marching algorithm.^11^ The shape channel and the material gradient are uploaded to the GPU as 3D textures and sampled along the ray paths. The marching ray shader is implemented in OpenGL Shading Language (GLSL) and High-Level Shading Language (HLSL), which computes the alpha weighted sum of all the points a ray encounters as it moves through the voxel field. To further speed up the shader, we use an offscreen low-resolution depth texture that contains the bounding box of the voxel field.^12^ The depth texture determines the starting points for the marching rays and concentrates the resolution of the rays within the volume. Finally, the marching step along a ray is slightly randomized to prevent the appearance of parallel artifacts.

Finally, a slicing algorithm was used to generate a stack of PNG slices similar to the steps used in the SlicerFab extension to a 3D Slicer. This process bypasses the traditional 3D printing method by sending the slice files directly to the 3D printer instead of a mesh file. Bitmaps are generated by slicing through the current volume rendering ROI and saving images as 32-bit PNG files.

Print parameters are explicitly defined to generate a slice through the volume rendering, which matched the parameters of the 3D printer. Since we are using a Stratasys J750,^13^ the X resolution is set to 600 DPI and the Y resolution is set to 300 DPI. The layer thickness is set to 0.027 mm and the slab thickness of the section box is set to 1 mm; thicker slabs will result in more opaque images and less noisy images, but some details may be blurred.

The printer used for this study is limited to six materials and a droplet resolution of 40 × 80 × 27 μm. Materials are mixed at the droplet level to create the appearance of thousands of colors at the macroscopic level. A material information database is constructed by characterizing material color properties matched with material-mixing ratios. Material-mixing ratios were created by dithering desired colors into droplet deposition descriptions for each distinct color in the 3D printer. Dithering was completed in Adobe Photoshop 2021 version 22.4.3, which sequentially processed all PNG images through a local perceptual Floyd Steinberg dithering algorithm limited to CMYKa. Finally, the resulting dithered 32-bit PNG files are sent to the printer.

Models created in Method 1 were printed on a Stratasys J750 3D printer (6 Material). VeroClear (RGD810) and VeroUltraClear were used as transparent materials, while for colors, VeroPureWhite (RGD837), VeroBlackPlus (RGD875), VeroYellow (RGD836), VeroCyan (RGD841), and VeroMagenta (RGD851) were used.

### Method 2—Mesh-based boundary surface model

**FIG. 3.**
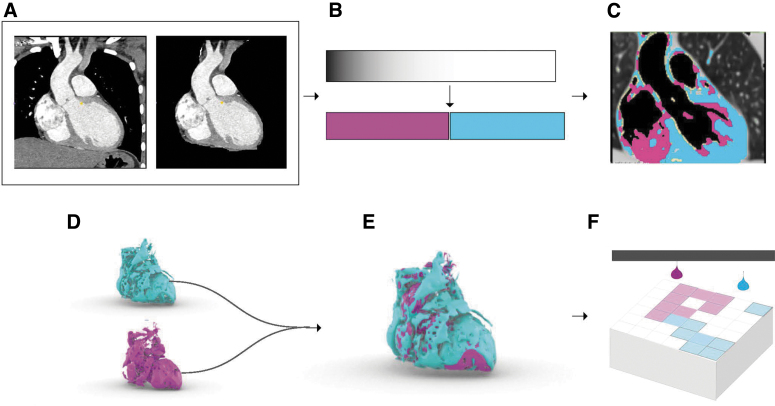
Mesh-based bounding surface modeling process workflow for modeling radiodensity. From the input DICOM data **(A)**, a mask is created to isolate the area of interest **(B)** from which a histogram is analyzed to parse the ranges of intensity values. **(C)** Multiple hulls are specified by thresholding segmentation according to the parsed ranges. **(D)** Separate mesh files are created for each individual segmentation and **(E)** combined in a printer-specific software for processing into a suitable file for **(F)** printing. A visualization of STL mesh models is equally divided into 2, 4, and 10 segmentations. STL models visually depict a loss in fidelity and volume from the source image as the number of segmentations increases.

An overview of our second method is shown in Figure 3. Using the open-source 3D Slicer Medical Imaging Software program DICOM files are loaded and viewed in a DICOM browser. First, the images are masked to eliminate the pixels, which lay outside the anatomy being segmented. Next, the images are segmented by using a threshold algorithm, which is defined by the set of ranges listed in Figure 2. In this process, each imaging data voxel is converted through a binary process to a solid, if the value of the voxel is within the specified range. This process was repeated for each desired threshold range, ensuring that the ranges are sequential with no overlap or numerical gap. The resulting segmentations were exported to STL using the OOTB export module in Slicer V10.4.2. This version of the 3D slicer processes the bounding mesh files utilizing the Flying Edges algorithm implemented by the VTK library.^14^ In this process, each voxel is converted to a volume bounded by triangulated facets, therefore, the number and size of the facets are dictated by the voxel size of the segmentation label map representation of the geometry. This process was repeated for each subsequent threshold range.

The resulting meshes were analyzed in the Rhino3D^15^ modeling software platform where the number of mesh faces, vertices, volume, and disconnected meshes were calculated for each mesh file. The resulting models created in Method 2 were fabricated using a commercially available Connex250 multimaterial 3D printer (Stratasys, Rehovot, Israel). Depending on the desired color range of the printed objects, high-modulus, Vero series, photopolymers of specific colors, and transparencies were employed as the constituent materials.

## Results

In all cases, we evenly divided, as a control, the given range of intensity values for each soft-tissue organ into 2, 4, and 10 regions. For the division of two regions, we printed the model in Vero Cyan and Vero Magenta, and for the division of four regions, we printed the model in Vero Cyan, Vero Magenta, Vero Yellow, and Tango Black. However, for the division of 10 regions, we printed the model in Vero Cyan, Vero Magenta, Vero Yellow, Tango Black, Vero Pure White, and Vero Clear with the remaining colors being achieved through the blending of these five base materials to create Red, Orange, Green, Blue, Violet.

Utilizing Method 1, a cross-sectional slice of each organ was rendered in each of the three division states noted above and bitmap printed. The results were studied visually and annotated by a Board-Certified Radiologist. It was observed that the emergence of anatomical features increased as the number of material divisions increased, which is shown by Table 1 to be preferred by clinicians. In the cardiac model, differentiation in pericardium, myocardium, and endocardium became present only with the use of 10 materials (Fig. 5). As a comparison of anatomies, the adolescent brain showed that the most distinct anatomical areas emerge from the addition of multiple materials, such as the Globus Pallidus critical to surgeries involving movement disorders, and the Occipital Horn of Lateral Ventricle where the presence of tumors can provide a massive challenge (Fig. 6).^16^

**Table 1.** Preference Table

	STL			Bitmap		
	2	4	10	2	4	10
Spatial resolution—radiologist						
Heart						XX
Kidney						XX
Brain						XX
Surgical planning—surgeon						
Heart						XX
Kidney						XX
Brain						XX
Patient communication—surgeon						
Heart						XX
Kidney						XX
Brain						XX
Clinical communication—radiologist/surgeon						
Heart						XX
Kidney						XX
Brain						XX

**FIG. 4.**
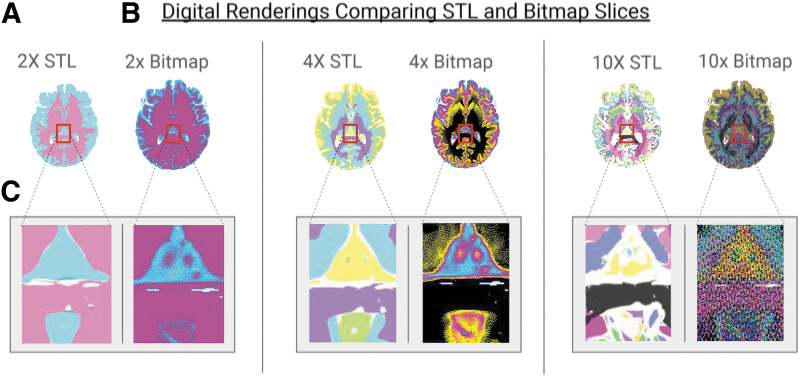
Digital renderings comparing STL and bitmap slices from the mesh-based bounding surface modeling method described in Figure 1 **(A)**, a cross-section of the STL model is shown for comparison to **(B)** a cross-section of the bitmap method described in Figure 1. **(C)** A small section is enlarged to demonstrate the boundaries of the STL process and gradients of the voxel process. The comparisons are repeated for the 4 and 10 segmentation models. Here, it is clear to see an erosion of material in the STL process with increased segmentation. Whereas the bitmap process maintains the volume while capturing more density information.

**FIG. 5.**
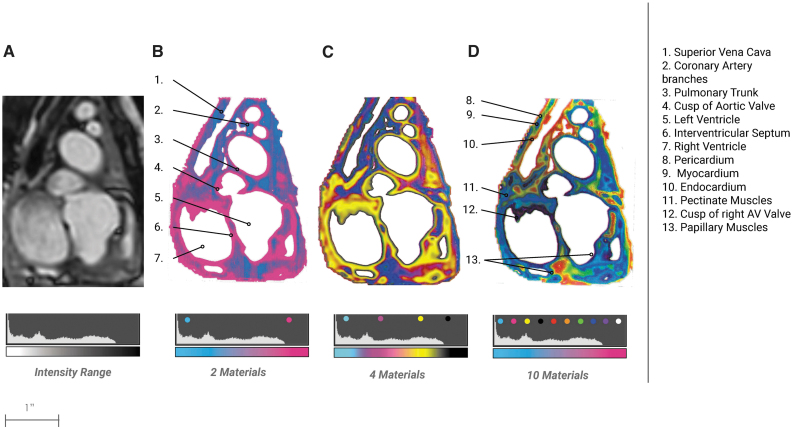
Cardiac Bitmap Progression. From the bitmap method described in Figure 1 **(A)** a DICOM image of a pediatric heart shows a cross-section. **(B)** A 3D printed plate is bitmap printed in two materials showing major structures and slight tissue differentiation **(C)** A bitmap printed plate is rendered in four materials. **(D)** A bitmap printed plate is rendered in 10 materials. It is clear to see the emergence of fine features and defined layers.

**FIG. 6.**
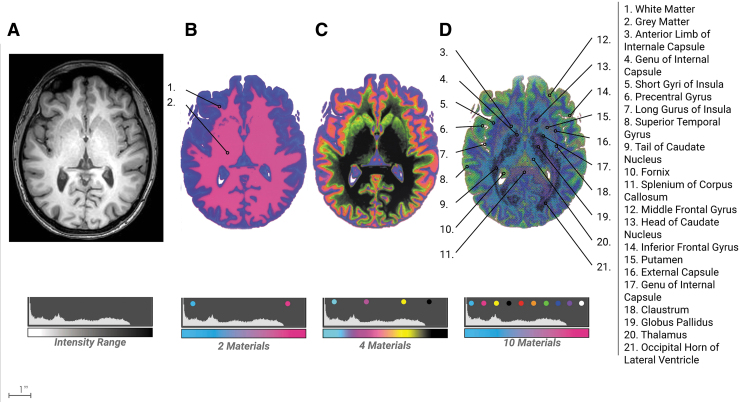
Neuro bitmap progression. From the bitmap method described in Figure 1 **(A)** a DICOM image of a pediatric brain showing an axial cross-section. **(B)** A 3D printed plate is bitmap printed in two materials showing major structures mostly limited to *gray* and *white* matter with slight tissue differentiation **(C)** A bitmap printed plate is rendered in four materials. **(D)** A bitmap printed plate is rendered in 10 materials. It is clear to see the emergence of many more structures critical to neurosurgical interventions.

**FIG. 7.**
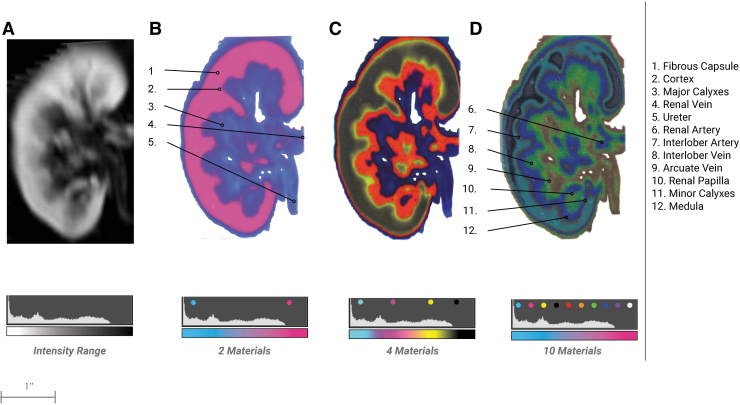
Kidney bitmap progression. From the bitmap method described in Figure 1 **(A)** a DICOM image of an adult kidney showing a coronal cross section. **(B)** A 3D printed plate is bitmap printed in two materials showing major structures and slight tissue differentiation. **(C)** A bitmap printed plate is rendered in four materials. **(D)** A bitmap printed plate is rendered in 10 materials. It is clear to see the emergence of many more structures critical to surgical interventions.

The division into two materials captured the distinction between gray and white matter, however, the division into 10 materials captured a substantial increase in anatomical features critical to surgical interventions, which was clearly identified by clinicians. The kidney model of two divisions clearly captured five major elements, however, the division into 10 materials allowed for the gradation between the medulla and cortex with a good definition of blood vessels and arteries at the intersection of the cortex and medulla (Fig. 7). In summary, in both methods, a larger number of threshold divisions yielded a higher visible emergence of anatomical features.

Method 1 and Method 2 were compared and digitally analyzed against the following metrics: (1) file size, (2) volume loss, and (3) disconnected elements.

### File size

**FIG. 8.**
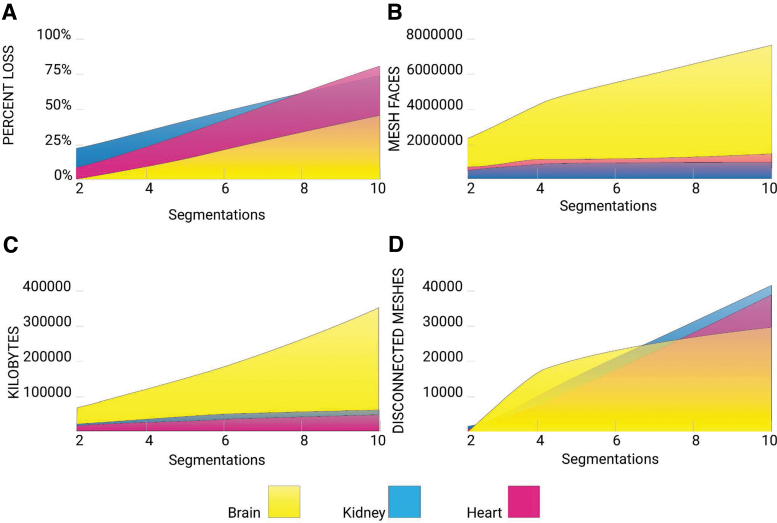
STL analysis. From the STL method described in Figure 3. **(A)** The percentage of volume being lost across increasing segmentation divisions are calculated and demonstrates a consistent increase across all anatomies. **(B)** The number of triangular mesh faces are calculated across increasing segmentation divisions. There is an unequal weight to the brain-based anatomy, which correlates to the kilobytes required to create the full STL file shown in **(C)**. **(D)** The number of disconnected meshes resulting from each segmentation division are calculated and show a relative consistency across all anatomies with slight variation respective to the brain model.

Method 2 uses thresholding and isosurface operations, which create triangular facets that scale in number with the number of internal boundaries; this quantity is large for volumetric representations of heterogeneous tissues. To correctly represent all the intensity values of medical CT data, ∼4000 separate mesh files (corresponding to the 12-bit discretization of Hounsfield unit radiodensity values typically recorded) would need to be defined and managed. An approximation of gradients could be created using several discrete regions of boundary surface representations with gradually changing colors or properties that could create the appearance of a gradient. However, based on our findings, this would result in print files reaching into the gigabytes. Although there are no defined file size limitations for 3D printing, larger files become unwieldy, slow to process, and computationally prohibitive. An overview of the File Size for the combined files of each anatomical division group is shown in Figure 8.

In contrast, the 32-bit PNG file size is governed by a 600 × 300 dpi resolution corresponding to the printer's native X and Y resolutions. Since slice thickness in biomedical imaging data sets can vary widely (usually ranging from 1 to 5 mm), our data sets were resliced at a 30-μm interval to match the 30-μm slice thickness of the multimaterial 3D printer used in this study. With these parameters, the largest combined voxel file, Brain 10, resulted in a total of 186 MB, whereas the mesh-based boundary surface model of Brain 10 totaled 363 MB for all combined files. However, using our method of voxel printing, an increase in segmentation divisions maintains the same memory allocation; the total file size for each Brain 2, 4, 10 voxel file set is 186 MB. This is because a voxel file size is not determined by the increase in pixel differentiation.

### Volume loss and disconnected meshes

Thresholding and isosurfacing operations involved in the creation of meshes as described in Method 2 introduce significant data loss. In all cases, this approach resulted in the creation of artificial boundaries, due to the nature of mesh-based boundary surface models that were not present in the native data and a decrease in the total volume of each boundary surface model with each additional division to the point where the remaining data were more than 80% decimated. These errors were a result of a combination of the segmentation and flying edges meshing algorithm, which attempts to create an isosurface using triangular facets around a grouping of similar density values. Interior voids introduced by the flying edges meshing algorithm are automatically filled by support material at print time, which is not optically clear and obscures print detail. In contrast, in our proposed voxel-based approach, where the dithered files are printed with the printer's native resolution, radiodensity information was accurately translated due to localized material mixing at the droplet level.

### Clinician survey

As a series of preliminary test cases, we compared the performance of both methods, the bitmap-based approach against the segmentation mesh-based boundary surface model 3D printing workflow, in the creation of a patient-specific 3D-printed model from three volumetric data sets (Fig. 4). The three data sets were: an adult patient with a right-sided kidney tumor (clear cell carcinoma), an adolescent patient with a brain tumor in the inferior frontal gyrus of the left cerebral hemisphere, and a pediatric patient with a single ventricle outlet congenital defect. The results of these models were given, blindly, to surgeons and radiologists to complete a qualitative survey. A pair of Board-Certified Medical Doctors, consisting of a Radiologist and a Surgeon, was given a set of models, which correlated with their respective specialties of Urologic Surgery Body Imaging, Pediatric Cardiothoracic Surgery/Pediatric Radiology, and Pediatric Neurosurgery/Pediatric Neuro Radiology. Each pair was given 2, 4, and 10 division models resulting from each of the two methods described above for a total of six models.

The survey asked clinicians to place a check next to the model which they preferred in response to four questions: (1) Which model has the highest accuracy of visual/spatial resolution? (2) Which model would you prefer for surgical planning? (3) Which model would you prefer to use in your communication with patients? and (4) Which model would you prefer in your communication with other clinicians? The survey results overwhelmingly indicated a preference for the bitmap models rendered in 10 divisions across all questions; see Table 1. These results are not intended to be construed as statistically significant; they do serve as positive preliminary data to be further explored in a full clinical trial.

## Discussion

The morphologic and radiodensity framework proposed in this study offers an approach enabling the assembly of physical visualizations of MRI and CT data. This method demonstrates how to realize more variation and differentiation in soft tissue by incorporating more material combinations into the PNG-based bitmap workflow. Each anatomical element contains unique differentiable features in soft tissue. This method can be easily tuned to focus on particular elements and relationships by modifying the number of materials.

Furthermore, the bitmap printing process exceeds the visual quality of mesh-based boundary surface model 3D printing workflows and methods described in the Introduction. Across all organs, we observe familiar issues related to file size, volume loss, and printability when using a mesh-based boundary surface modeling workflow. However, the requirements of each anatomy differ slightly. The brain model proved to be the most computationally expensive, generating large files and prolonged processing time due to the large number of mesh faces required to capture the differentiation as densities increased. Concerning the total volume lost and the number of disconnected elements, the three anatomies tracked closely, with the brain showing slightly fewer issues than the cardiac and kidney models.

### File size

As shown in Figure 8, the mesh-based boundary surface modeling process generates consistently larger file sizes as the number of segmentation divisions increases. For these multiple segmentations, a threshold algorithm is traditionally used to create an enclosure for every range of radiodensity desired, which consequently increases the vertex, face, and file size of the new data set by roughly a factor of 10 with each additional segmentation compared with the source file. By using our patent-pending voxel printing process, the method presented in this study allows for reduced computational expense, by eliminating segmentations of the data sets compared with methods using boundary representations. Bitmap files for each segmentation maintained a consistent file size, however, additional divisions and layers of imaging data incur slight increases in file size, which is limited to double-digit kilobyte increases per additional slice. This is because the voxel method bypasses the generation of boundary representations, and works directly from the source images.

### Volume loss

As additional segmentations are created, the enclosed area shrinks due to the fact that the meshing algorithm does not enforce coincident faces between different segmentation, thereby allowing for gaps to form between multiple segmentations. This creates a loss of volume by roughly a factor of 10 with each additional segmentation compared with the source file. This volume decrease is mitigated through our voxel printing method. The process of creating a mesh-based surface boundary from a segmentation, utilizing the flying edges meshing algorithm, is to approximate a polygonal surface from the isosurface segmentation based on 256 possible polygon configurations.^17^ Similar to the marching cubes algorithm, the flying edges algorithm uses the isosurface segmentation as a guide but operates upon the base voxel data inherited from source imaging. Boundary representations label voxels as either solid or void (inside or outside), which results in pixelation and aliasing along the boundaries. The possibility of three dimensionally supersampling boundaries may avoid this effect, but this has not yet been accomplished.

Therefore, our mesh-based boundary surface process resulted in a 50–80% reduction, depending on anatomy, in volume from the source data using only 10 density threshold segmentations or 0.25% of the total amount of divisions possible. Our methods allow for the 3D printing of medical images with minimal information loss, compared with the mesh-based boundary surface 3D printing method. As an example, medical images must be converted into a 3D volumetric data structure, visualized as a volume rendering, where every pixel in the image is mapped to a voxel or volumetric pixel. Medical images are typically collected at a resolution of 1–5 mm. The generated volumetric renderings, therefore, contain millions of voxels, making any processing on this data set computationally expensive. The isosurface extraction process inherent to the generation of mesh-based boundary surface files leads to a surface mesh composed of polygons that, despite the number of segmentations, fails to capture the fine details of the source images.

### Gradients

Boundary surfaces cannot, by definition, represent gradients; they can only approximate them by concatenating several regions with successive transitions. In principle, one could concatenate so many surfaces that the gradient appears smooth at the resolution of the printer, but this would require a very large number of surfaces. In this process, each region would be processed as a unique file, which aside from the complications related to managing such a large file repository, would require gigabytes of memory to process and fabricate.

## Conclusion

The current representational framework that the majority of, if not all, digital modeling tools employ utilizes a boundary surface file format. This paradigm has proven inadequate when trying to express the granular or hierarchical structure of more complex natural materials. With the arrival of recent bitmap printing techniques, we can now produce high-resolution and high-contrast objects, which display gradual material transitions throughout their volume. This article demonstrates that a voxel or raster-based process is more suited to accurately represent soft tissue from radiological images. Furthermore, we provide a method for replicating more of the material definitions described in radiological images. This method improves upon previous attempts to bitmap print utilizing two materials by simplifying the process and incorporating six materials. The benefits of this workflow include: a simplified method for multimaterial bitmap printing, precise graduated control over the material distribution at multiple scales within a 3D printed volume, and a more accurate representation of anatomical features in soft tissue.

Our major findings indicate that a multimaterial bitmap representation of soft tissue shows finer levels of variations in material descriptions and outperforms the predominant surface mesh-based process for 3D printing. Our other finding supports previous discoveries that mesh-based boundary surface modeling methods are inadequate when printing multiple variations and gradients in soft tissue. This method does not accurately replicate tissue densities specific to the patient and defines a limitation of this method for replicating biological tissue. This further supports the claims of bitmap printing's ability to translate morphological and radiodensity data from medical images without a loss or alteration of information.

The implications of this technique allow for new creative applications for presurgical planning models involving soft tissue. As laparoscopic surgeries, without haptic feedback, continue to replace open procedures, the current and future generation of surgeons have less hands-on experience. This inexperience has led to errors and complications relating to excessive force.^18^ In these cases, our density mimetic models can bridge this knowledge gap through benchtop training. Furthermore, for tumor resections in soft tissue organs such as the kidney and brain, the ability to volumetrically visualize the anatomy has the potential to decrease margin rates to improve the removal of cancers by giving surgeons an accurate structural understanding of tumor morphology with surrounding tissues. Finally, the bitmap printing workflow presented in this study, has exciting implications for developing fine control over mechanical properties, such as compliance, in the same way that we have achieved fine control over color. This is critical to stent and valve placement surgeries, where vascular compliance is still highly unpredictable, leading to complications with implantable expanding devices.

## References

[1] Chepelev L, Wake N, Ryan J, *et al.* Radiological Society of North America (RSNA) 3D printing Special Interest Group (SIG): Guidelines for medical 3D printing and appropriateness for clinical scenarios. 3D Print Med 2018;4:11. 30649688 10.1186/s41205-018-0030-y PMC6251945

[2] Qin Y, Qunfen Q, Scott P, *et al.* Status, comparison, and future of the representations of additive manufacturing data. Comput Aided Des Appl 2019;111:44–64.

[3] Yan Q, Dong H, Su J, *et al.* A review of 3D printing technology for medical applications. Proc Est Acad Sci Eng 2018;4:729–742.

[4] Hongo F, Fujihara A, Inoue Y, *et al.* Three-dimensional-printed soft kidney model for surgical simulation of robot-assisted partial nephrectomy: A proof-of-concept study. Int J Urol 2021;28:870–871. 33821541 10.1111/iju.14560

[5] Melnyk R, Ezzat B, Belfast E, *et al.* Mechanical and functional validation of a perfused, robot-assisted partial nephrectomy simulation platform using a combination of 3D printing and hydrogel casting. World J Urol 2020;38:1631–1641. 31679063 10.1007/s00345-019-02989-z PMC7730938

[6] Bianchi L, Barbaresi U, Cercenelli L, *et al.* The impact of 3D digital reconstruction on the surgical planning of partial nephrectomy: A case-control study. Still time for a novel surgical trend? Clin Genitourin Cancer 2020;18:e669–e678. 32354617 10.1016/j.clgc.2020.03.016

[7] Jacobson N, Smith L, Brusilovsky J, et al. Defining soft tissue: Bitmap printing of soft tissue for surgical planning. 3D Print Addit Manuf 41:56–60. 10.1089/3dp.2021.0141 PMC9809978 36654967

[8] Harper G, Jain SH, Pories S. The Soul of a Doctor: Harvard Medical Students Face Life and Death. Chapel Hill, South Carolina: Algonquin Books, 2012.

[9] Hosny A, Keating SJ, Dilley JD, *et al.* From improved diagnostics to presurgical planning: High-resolution functionally graded multimaterial 3D printing of biomedical tomographic data sets. 3D Print Addit Manuf 2018;5:103–113.

[10] Fedorov A, Beichel R, Kalpathy-Cramer J, *et al.* 3D Slicer as an image computing platform for the Quantitative Imaging Network. Magn Reson Imaging 2012;30:1323–1341. 22770690 10.1016/j.mri.2012.05.001 PMC3466397

[11] Hadwiger M, Laura F, Rezk-Salama C, *et al.* Interactive volume exploration for feature detection and quantification in industrial CT data. IEEE Trans Vis Comput Graph 2008;14:1507–1514. 18989003 10.1109/TVCG.2008.147

[12] Cline HE, Dumoulin CL, Hart HR Jr, *et al.* 3D reconstruction of the brain from magnetic resonance images using a connectivity algorithm. Magn Reson Imaging 1987;5:345–352. 3695821 10.1016/0730-725x(87)90124-x

[13] GrabCAD. Guide to voxel printing. Available at: https://help.grabcad.com/article/230-guide-to-voxel-printing?locale=en (last accessed February 16, 2021).

[14] Hopkinson N, Hague R, Dickens P. Rapid Manufacturing: An Industrial Revolution for the Digital Age. Hoboken, New Jersey: John Wiley & Sons, 2006.

[15] McNeel R & Associates. Rhinoceros 3D. Available at: https://www.rhino3d.com/ (last accessed May 28, 2021).

[16] Cikla U, Swanson KI, Tumturk A, *et al.* Microsurgical resection of tumors of the lateral and third ventricles: Operative corridors for difficult-to-reach lesions. J Neurooncol 2016;130:331. 27235145 10.1007/s11060-016-2126-9 PMC5090015

[17] Dietrich CA, Scheidegger CE, Schreiner J, *et al.* Edge transformations for improving mesh quality of marching cubes. IEEE Trans Vis Comput Graph 2009;15:150–159. 19008562 10.1109/TVCG.2008.60

[18] Abiri A, Pensa J, Tao A, *et al.* Multi-modal haptic feedback for grip force reduction in robotic surgery. Sci Rep 2019;9:5016. 30899082 10.1038/s41598-019-40821-1 PMC6428814

